# PCGF6 regulates stem cell pluripotency as a transcription activator via super-enhancer dependent chromatin interactions

**DOI:** 10.1007/s13238-019-0629-9

**Published:** 2019-04-30

**Authors:** Xiaona Huang, Chao Wei, Fenjie Li, Lumeng Jia, Pengguihang Zeng, Jiahe Li, Jin Tan, Tuanfeng Sun, Shaoshuai Jiang, Jia Wang, Xiuxiao Tang, Qingquan Zhao, Bin Liu, Limin Rong, Cheng Li, Junjun Ding

**Affiliations:** 1 RNA Biomedical Institute, Sun Yat-Sen Memorial Hospital, Zhongshan School of Medicine, Sun Yat-Sen University, Guangzhou, 510080 China; 2 Center for Stem Cell Biology and Tissue Engineering, Key Laboratory for Stem Cells and Tissue Engineering, Ministry of Education, Sun Yat-Sen University, Guangzhou, 510080 China; 3 Department of Cell Biology, Zhongshan School of Medicine, Sun Yat-sen University, Guangzhou, 510080 China; 4 Program in Stem Cell and Regenerative Medicine, The Third Affiliated Hospital of Sun Yat-sen University, Zhongshan School of Medicine, Sun Yat-Sen University, Guangzhou, China; 5 Center for Bioinformatics, School of Life Sciences, Peking University, Beijing, 100871 China; 6 Department of Spine Surgery, The Third Affiliated Hospital of Sun Yat-sen University, Guangzhou, 510630 China; 7 Department of Histology and Embryology, School of Basic Medical Sciences, Guangzhou Medical University, Guangzhou, 511436 China

**Keywords:** PCGF6, Polycomb group, super-enhancer, 3D chromatin, pluripotency

## Abstract

Polycomb group (PcG) ring finger protein 6 (PCGF6), though known as a member of the transcription-repressing complexes, PcG, also has activation function in regulating pluripotency gene expression. However, the mechanism underlying the activation function of PCGF6 is poorly understood. Here, we found that PCGF6 co-localizes to gene activation regions along with pluripotency factors such as OCT4. In addition, PCGF6 was recruited to a subset of the super-enhancer (SE) regions upstream of cell cycle-associated genes by OCT4, and increased their expression. By combining with promoter capture Hi-C data, we found that PCGF6 activates cell cycle genes by regulating SE-promoter interactions via 3D chromatin. Our findings highlight a novel mechanism of PcG protein in regulating pluripotency, and provide a research basis for the therapeutic application of pluripotent stem cells.

**Electronic supplementary material:**

The online version of this article (10.1007/s13238-019-0629-9) contains supplementary material, which is available to authorized users.

## Introduction

Embryonic stem cells (ESCs) are derived from the inner cell mass of blastocyst stage embryos, and theoretically have the capacity to differentiate into all cell types of the three germ layers, thus are widely used for pluripotency research (Evans and Kaufman, 1981; Martin, 1981). The pluripotency of ESCs is maintained primarily by a network including master transcription factors OCT4, SOX2 and NANOG (Ivanova et al., 2006; Chen et al., 2008; Kim et al., 2008), along with multiple protein complexes for both developmental gene repression and stemness gene activation. Polycomb groups (PcG) and Trithorax groups (TrxG) proteins are respectively the well-known repressor and activator complexes at present (Jaenisch and Young, 2008; Schuettengruber and Cavalli, 2009; Schuettengruber et al., 2011). PcG proteins were first identified in *Drosophila melanogaster* as regulators of the *Hox* (homeotic) cluster genes, and subsequently shown to be essential for developmental gene regulation via chromatin modification (Lewis, 1978; Schwartz and Pirrotta, 2013). The PcG proteins are broadly classified into two complexes called Polycomb repressive complex 1 (PRC1) and Polycomb repressive complex 2 (PRC2). PRC2 contains a histone H3 lysine 27 (H3K27) methyltransferase (Cao et al., 2002; Margueron and Reinberg, 2011), while PRC1 contains a histone E3 ubiquitin ligase that catalyzes mono-ubiquitylates histone H2A at position 119 (H2AK119ub1) (de Napoles et al., 2004; Wang et al., 2004; Cao et al., 2005). In addition, PRC1 also includes RING1A or RING1B, CBX (chromobox homolog), PHC (polyhomeotic homolog) proteins, and paralogs of PCGF (Polycomb group ring finger, PCGF1–6) (Francis et al., 2001).

Polycomb group factor 6 (PCGF6), also known as MBLR (MEL18 and BMI1-like RING finger protein), is a member of the Polycomb group family, which canonically acts as a transcription repressor (Akasaka et al., 2002). Recent advances improved our understanding that *Pcgf6* can be a substitute of *Sox2* in the generation of germline-competent induced pluripotent stem cells (iPSCs), and it also has the function of activating pluripotency genes to maintain ESC pluripotency (Zdzieblo et al., 2014; Yang et al., 2016). However, the mechanism of PCGF6-mediated transcriptional activation remains to be elucidated.

Previous studies show that PCGF6 is enriched in the promoters of pluripotency-associated genes like *Oct4*, *Sox2*, *Nanog*, *Lin28* and *Myc* (Yang et al., 2016). Knockdown of *Pcgf6* downregulates these pluripotency genes (*Oct4*, *Sox2* and *Nanog*) and leads to cell differentiation (Hu et al., 2009). Consistently, overexpression of *Pcgf6* increased the expression of *Oct4*, *Sox2* and *Nanog* (Yang et al., 2016). These pluripotency factors regulate specific gene expression by interacting with the upstream enhancer elements (Buecker et al., 2014), which can be classified into the typical enhancers (TEs) and super-enhancers (SEs). Compared with TEs, SEs are large clusters of transcriptional enhancers and have been shown to activate the expression of pluripotency genes including *Oct4*, *Sox2* and *Nanog* in ESCs (Hnisz et al., 2013; Whyte et al., 2013). Therefore, we hypothesized that PCGF6 activates the pluripotency factors through SEs.

The three dimension (3D) chromatin structure is considered to regulate gene expression via forming active or repressive transcription domains by chromosome-structuring proteins like CTCF, YY1 and cohesin (Bickmore, 2013; de Graaf and van Steensel, 2013; de Laat and Duboule, 2013; Weintraub et al., 2017). Recent studies show that chromatin 3D structure enables the SEs to interact with distal promoters of specific genes (Ji et al., 2016). However, it is not clearly understood whether PCGF6 regulates pluripotency via this SE-dependent 3D chromatin interaction. Importantly, OCT4, SOX2 and NANOG (OSN) are highly enriched in the SE regions (Hnisz et al., 2013; Whyte et al., 2013; Ji et al., 2016). Forced expression of reprogramming factors including OCT4, SOX2, and NANOG during somatic cell reprogramming is accompanied by chromatin remodeling (Krijger et al., 2016). Therefore, it is important to test whether PCGF6 coordinates with pluripotency factors regulate pluripotency via super-enhancer dependent 3D chromatin interactions.

The role of PCGF6 in cell fate decision is well established, wherein it not only represses developmental genes as a component of the PRC1 complex, but also activates pluripotency genes. Herein, our study aim to elucidate the mechanism of PCGF6 regulating pluripotency by activating the expression of proliferation genes such as *Ccnd3* and *Polr3gl* via SEs. Moreover, we also found those genes are directly regulated by PCGF6 mediated 3D chromatin interactions. We thus provide potential new insights into the mechanism of PcG components.

## Results

### *Pcgf6* is required for the establishment and maintenance of pluripotency

**Figure 1:**
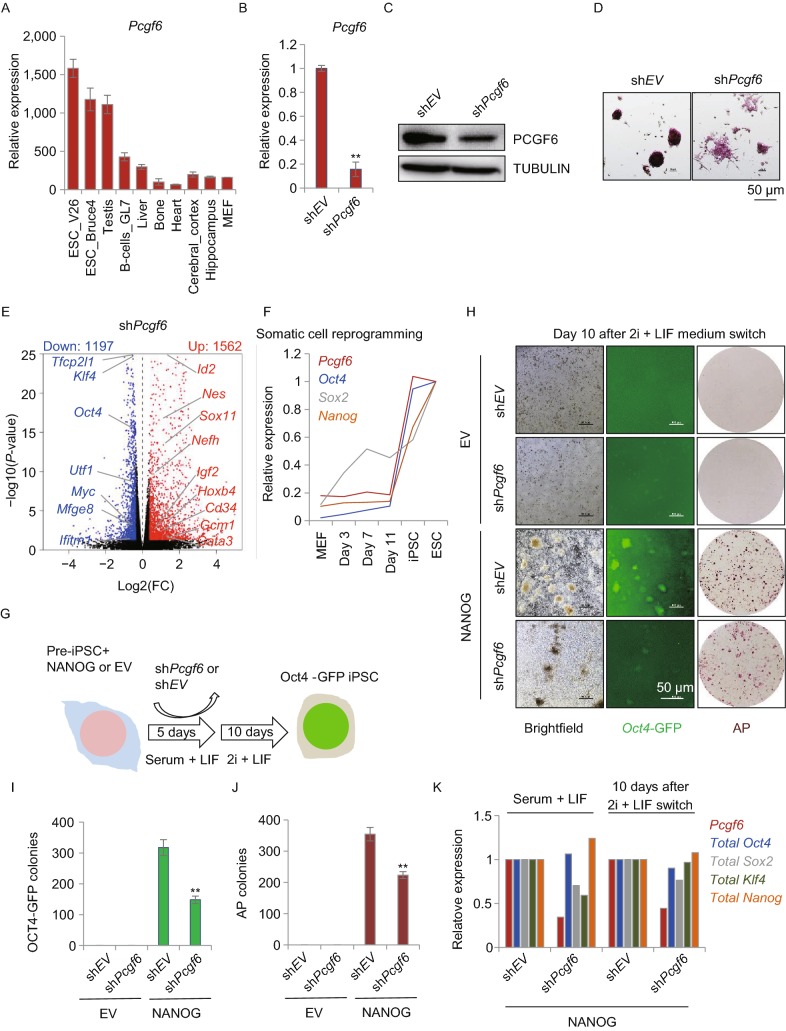
**Effects of PCGF6 on the maintenance and establishment of pluripotency in mESCs**. (A) The expression of PCGF6 in different tissues. (B) Knockdown efficiency of *Pcgf6* in mESCs was validated by real-time quantitative PCR (qPCR). Data are presented as mean ± SD from three independent replicates. **P < 0.01 compared with control cells. (C) Knockdown efficiency of *Pcgf6* in mESCs was validated by Western blot. (D) Morphology of *Pcgf6* knockdown mESCs with AP staining in the day 5, respectively. Scale bar represents 50 μm. (E) Volcano map of the RNA-Seq expression data from empty vector and *Pcgf6* knockdown mESCs. 1.3 fold change and *P* < 0.05 was significantly. (F) The dynamic expression of genes *Pcgf6*, *Oct4*, *Sox2* and *Nanog* during somatic cell reprogramming. The data was analyzed using the microarray data for gene expression from GSE19023 (Heng et al., 2010). (G) Schematic overview of the reprogramming process using stable pre-iPS cell lines and medium switch from serum/LIF to N2B27/2i/LIF. (H) The knockdown of *Pcgf6* significantly reduces the reprogramming efficiency. Bright field (left), GFP field (middle) and AP staining (right) in day 10 after 2i + LIF medium switch. Scale bar represents 500 μm. (I) Quantification of the numbers of *Oct4*-GFP colonies at day 10 of N2B27/2i/LIF treatment. Data are presented as mean ± SD from three independent replicates. **P < 0.01 compared with control cells. (J) Quantification of the numbers of colony formation at day 10 of N2B27/2i/LIF treatment. Data are presented as mean ± SD from three independent replicates. **P < 0.01 compared with control cells. (K) Total expression of *Oct4*, *Sox2*, *Nanog*, *Klf4* and *Pcgf6* in day 0 of serum/LIF and day 10 of N2B27/2i/LIF treatment. Data are presented as mean ± SD from three independent replicates

To investigate the role of *Pcgf6* on pluripotency maintenance of mouse embryonic stem cell (mESC), we first analyzed the expression of *Pcgf6* in different tissues. *Pcgf6* showed the highest expression in the murine ESCs, ESC_V26 and ESC_Bruce4, compared to that in other tissues like liver, heart and MEF, etc. (Fig. 1A), indicating a vital role in mESCs. To explore the potential functions of *Pcgf6*, we performed loss-of-function experiments in mESCs. The knockdown efficiency was validated by RT-qPCR and Western blot, respectively (Fig. 1B and 1C). Alkaline phosphatase staining analysis showed an obvious differentiation phenotype after *Pcgf6* knockdown (Fig. 1D), which suggested that *Pcgf6* regulates the pluripotency of mESCs. To further dissect the underlying molecular mechanism, we analyzed the transcriptomes by carrying out RNA-Seq assay in *Pcgf6* knockdown and control mESCs to investigate the transcription alteration. The results showed that a total of 1197 genes were significantly downregulated while 1562 genes were significantly upregulated in *Pcgf6* knockdown cells (Fig. 1E; Table S3). A majority of pluripotency associated genes like *Oct4* and *Klf4* were downregulated, whereas the upregulated genes cover a portion of genes involved in development, such as *Gcm1, Gata3* and *Hand1* (trophectoderm), *Hoxb4, Cd34* and *T* (mesoderm), *Pax3, Nestin* and *Sox11* (ectoderm), and *Gata6* and *Sox17* (endoderm) (Fig. S1A–E). Taken together, *Pcgf6* globally influences the pluripotency transcription network in mESCs.

Previous studies show that *Pcgf6* is required for the somatic cell reprogramming (Zdzieblo et al., 2014; Yang et al., 2016). However, it is still unclear which stage of reprogramming does *Pcgf6* affects. These studies prompted us to further evaluate the role of *Pcgf6* on establishment of pluripotency. During the somatic cell reprogramming, we found that *Pcgf6* was slightly upregulated from day 0 (MEF) till day 11, and dramatically increased thereafter to the iPSC stage (Fig. 1F). These results indicate that *Pcgf6* is mainly activated in the later stage of somatic cell reprogramming. To verify this hypothesis, we degenerated the expression of *Pcgf6* in pre-iPSCs expressing an *Oct4*-GFP reporter gene and overexpressing the reprogramming factor *Nanog* (Fig. 1G). The proportion of GFP+ undifferentiated colonies in the *Pcgf6* knockdown group was only 46.6% of that in the control group after 10 days of culture in N2B27/2i/LIF medium (Fig. 1H and 1I). The percentage of undifferentiated colonies by alkaline phosphatase (AP) staining analysis was 63% of that in control group (Fig. 1J). In addition, the total mRNA expression of pluripotency genes such as *Sox2* and *Klf4* was downregulated after *Pcgf6* knockdown (Fig. 1K). Taken together, *Pcgf6* is important for both ESC maintenance and late stage establishment of pluripotency.

### PCGF6 activates the expression of genes associated with pluripotency

**Figure 2:**
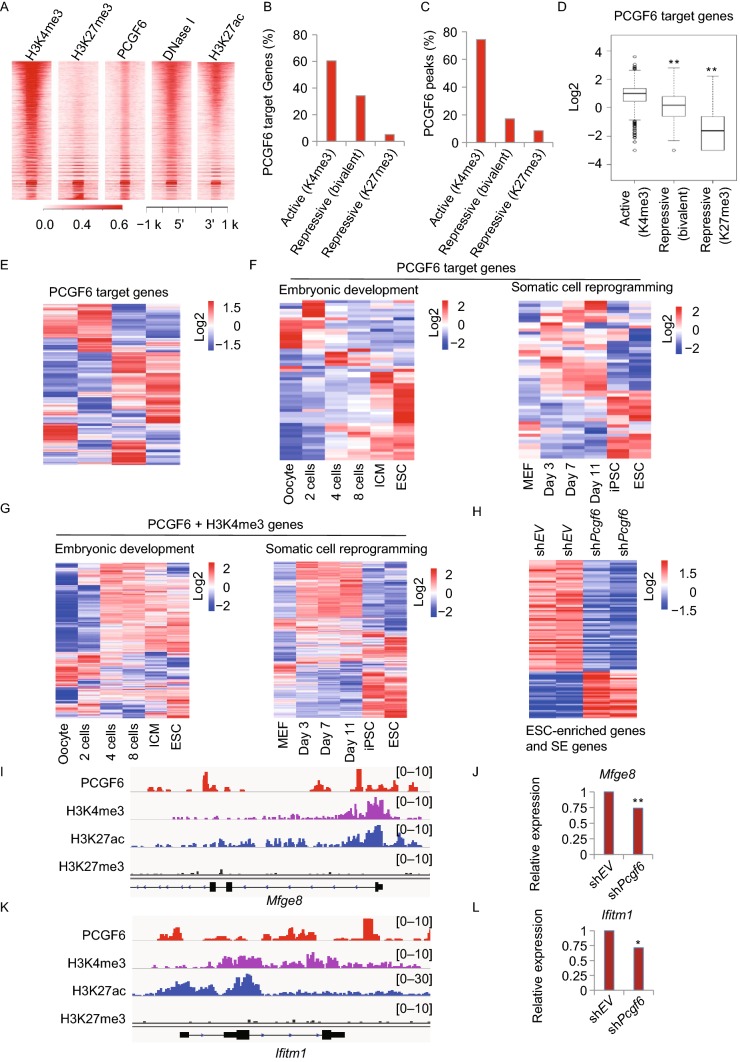
**Transcription activation function of PCGF6**. (A) Heatmaps of PCGF6 binding loci are sorted by the active marker H3K4me3 and the repress marker H3K27me3. H3K27ac is an active marker. DNase I is an open chromatin markers. (B and C) Percentage of PCGF6 targeted peaks (B) and genes (C) in active (K4me3) regions, repressive (bivalent) regions, and repressive (K27me3) regions. (D) Box plot of gene expression of PCGF6 targeted genes in mESCs. **P < 0.01 compared with active (K4me3) group. (E) Relative expression of PCGF6 targeted genes in Pcgf6-depleted group compared with empty vector group. Data are presented in two independent replicates. (F) Heatmaps showing the dynamic expression of PCGF6 targeted genes during embryonic development and somatic cell reprogramming. (G) Heatmaps shows the dynamic expression of PCGF6/H3K4me3 co-binding genes during embryonic development and somatic cell reprogramming. (H) Relative expression of ESC-associated genes from the RNA-Seq data. The gene list generated from ESC-enriched genes (Ben-Porath et al., 2008) and SE associated genes (Whyte et al., 2013). (I–L) The enrichment of PCGF6, H3K27ac, H3K4me3 and H3K27me3 at the locus of *Mfge8* (I) and *Ifitm1* (K). Relative expression of *Mfge8* (J) and *Ifitm1* (L) after *Pcgf6* knockdown were presented. **P < 0.01 compared with control cells. *P < 0.05 compared with control cells

Previous studies have shown that PCGF6 is a transcriptional activator (Yang et al., 2016). Based on this above, and our finding that *Pcgf6* depletion downregulated a majority of pluripotency genes (Fig. 1SA–E), we hypothesized that PCGF6 maintains ESC pluripotency via transcriptionally activating the relevant genes. To further dissect if PCGF6 activates gene expression directly, we analyzed the ChIP-Seq data by Yang et al. (2016) to identify the genome-wide binding sites of PCGF6. PCGF6 was highly enriched in the regions with active chromatin marker (H3K4me3 alone), compared to those with repressive chromatin marker (H3K27me3 alone) (Fig. 2A), and we defined these regions as active (K4me3) regions. The active regions were also positive for other open chromatin markers like H3K27ac and DNase I (Fig. 2A), indicating strong transcriptional activation feature of these regions. As a well-known component of Polycomb complex, PCGF6 is also enriched in the regions with chromatin marker “H3K4me3 + H3K27me3” or “H3K27me3 alone” defined as repressive (bivalent) or repressive (K27me3) regions respectively according to Yang et al, (2016) (Fig. 2A). Furthermore, the proportion of PCGF6 peaks and target genes in the active (K4me3) regions were significantly higher than that in the repressive (bivalent) or repressive (K27me3) regions (Fig. 2B and 2C; Table S4), as were their expression in mESCs (Fig. 2D). Taken together, these results verified that PCGF6 transcriptionally activates its target genes in mESCs. To further demonstrate the direct effect of PCGF6 on the active regions, we analyzed the expression of the PCGF6 target genes after *Pcgf6* depletion. A large portion of these genes were downregulated after *Pcgf6* knockdown, further verifying an activation function in addition to the canonical repressive function of PCGF6 (Fig. 2E).

To determine any potential role of the genes targeted by PCGF6 in regulating pluripotency of mESCs, we evaluated their expression patterns during embryonic development and somatic cell reprogramming (Fig. 2F). Majority of these genes were highly expressed in multiple stages, especially in the stages of inner cell mass (ICM) and ESCs during embryonic development, and in both ESCs and iPSCs during somatic cell reprogramming (Fig. 2F), indicating these genes are required for pluripotency regulation. In addition, more than 90% of the PCGF6 target genes enriched in the active region but not in the repressive region were upregulated from the 2-cell to blastocyst stage during embryonic development (Fig. 2G). Consistently, more than 60% of these genes showed the highest expression at the iPSC stage during somatic cell reprogramming (Fig. 2G). The remaining genes were highly expressed from day 3 to day 11 and then downregulated in the later stage, indicating an important role of PCGF6 during the somatic cell reprogramming (Fig. 2G). Previous studies have defined ESC-enriched genes and SE-associated genes, which are important for the maintenance of pluripotency in mESCs (Ben-Porath et al., 2008; Whyte et al., 2013). We found that 69 of ESC-enriched genes and SE-associated genes were significantly downregulated after *Pcgf6* knockdown (Fig. 2H). For example, PCGF6 targets the promoter regions of *Mfge8 and Ifitm1*, where H3K4me3 is highly enriched while H3K27me3 is absent, and both genes were significantly downregulated in response to *Pcgf6* knockdown (Fig. 2I–L). Taken together, our results indicate that *Pcgf6* is required for the expression of pluripotency-associated genes.

### PCGF6 positively regulates pluripotency genes through super-enhancers

**Figure 3:**
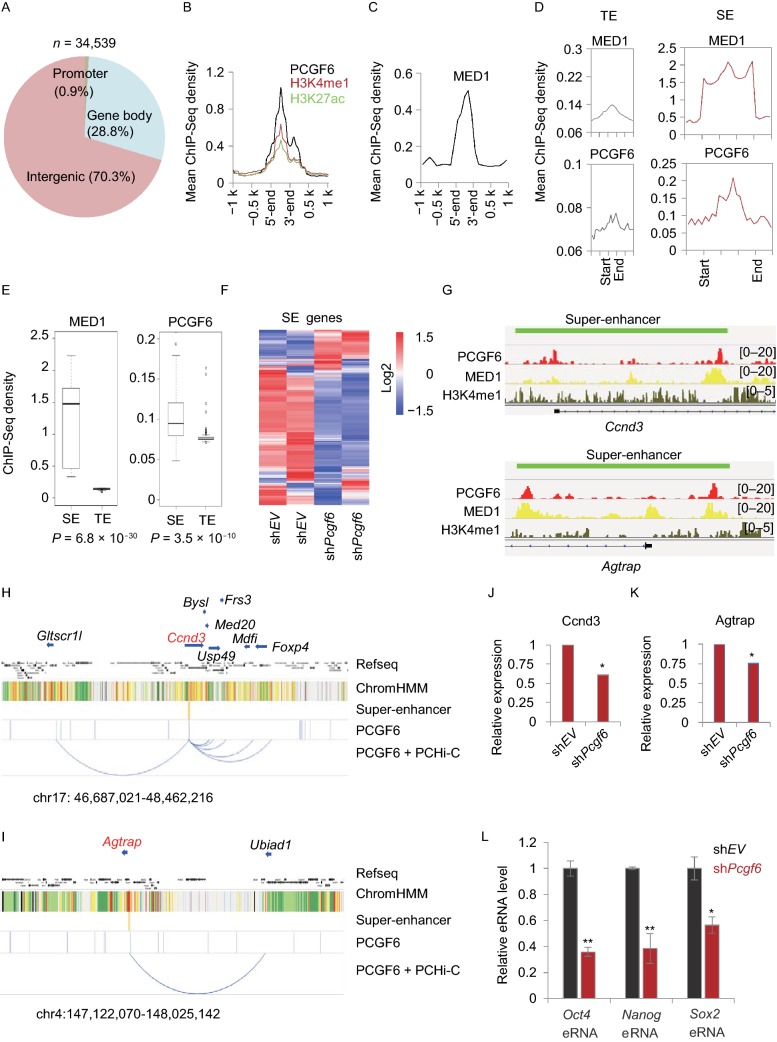
**PCGF6 positively regulates pluripotency genes via super-enhancers**. (A) Distribution of PCGF6 binding sites at promoter (−3 kb to +3 kb), gene body, and intergenic regions. (B) Average ChIP-Seq density of H3K4me1 and H3K27ac near the PCGF6 peak center. (C) Average ChIP-Seq density of MED1 near the PCGF6 peak center. (D) The average ChIP-Seq density of PCGF6 and MED1 in typical enhancers (TEs) and super enhancers (SEs). (E) Box plot of MED1 (left) and PCGF6 (right) ChIP-Seq density (reads per million reads per base) at the TE and SE regions. (F) Relative expression of SE-associated genes in *Pcgf6*-depleted group compared with empty vector group. The genes that closest to SEs were selected. (G) The enrichment of PCGF6, MED1, and H3K4me1 at the SE region of *Ccnd3* and *Agtrap*. (H–K) Promoter-associated interactions of *Ccnd3* (H) and *Agtrap* (I) derived from promoter capture Hi-C reads. Relative expression of *Ccnd3* (J) and *Agtrap* (K) after *Pcgf6* knockdown were presented. The regions and genes above are closest to SE and PCGF6 binding site. *P < 0.05 compared with control cells. (L) eRNA expression of Oct4, Sox2 and Nanog SEs after 5 days of *Pcgf6* knockdown. Data are presented as mean ± SEM (*n* = 3). *P < 0.05 compared with control cells. **P < 0.01 compared with control cells

To further dissect the molecular mechanism underlying PCGF6-mediated regulation of pluripotency, we tracked the distribution of PCGF6 binding sites in the entire genome. A total of 34,539 peaks were identified, 70.3% of which were distributed in the intergenic regions, and only 0.9% in the promoter regions (Fig. 3A). Since transcriptional enhancers mainly localize to the intergenic regions, we hypothesized that PCGF6 regulates transcription through enhancer elements. Consistent with this, the PCGF6 binding regions were significantly enriched in the SE regions with active histone modification H3K4me1 and H3K27ac (Fig. 3B). Moreover, MED1 was also highly enriched in the center of PCGF6 binding regions (Fig. 3C). In addition, the core pluripotency factors, including OCT4, SOX2 and NANOG (OSN), which usually co-localize at the SE regions in mESCs, were also co-enriched with PCGF6 (Fig. S2A), indicating an enhancer dependent regulation pathway of PCGF6. Although PCGF6 was enriched in both the TE regions and SE regions, the enrichment of PCGF6 in SE regions are significantly higher than that in TE regions (Fig. 3D and 3E).

Since the SEs frequently regulate the cell fate-related genes and maintain their high expression in mESCs, we surmised that absence of PCGF6 would downregulate these genes. Indeed, *Pcgf6* knockdown downregulated most SE-associated genes while a few were upregulated (Fig. 3F), further underscoring that PCGF6 regulates pluripotency via an SE-related pathway. Moreover, the significantly downregulated genes included *Tet2, Klf4* and *Oct4* (Fig. S2B), which are required for both establishing and maintaining pluripotency (Fig. S2C). As an example, *Ccnd3* and *Agtrap* are the adjacent genes of SE targeted by PCGF6, which indicates that they might be SE-regulated genes. PCGF6 co-localized with H3K4me1, H3K27ac and MED1 in SE regions upstream of *Ccnd3* and *Agtrap*, which were significantly downregulated after *Pcgf6* knockdown (Figs. 3G, 3J and 2K), indicating PCGF6 regulates the expression of pluripotency genes through SEs. Moreover, promoter capture Hi-C analysis showed that PCGF6-targeted SEs mediated the promoter-promoter interactions of the *Ccnd3* or *Agtrap* with their adjacent genes, respectively (Fig. 3H and 3I). To further dissect the relationship between PCGF6 and SEs, we detected the expression of eRNA and found that depletion of PCGF6 decreased the expression of eRNAs that arise from OSN SEs (Fig. 3L). These results illustrate an important role of PCGF6 in activating pluripotency genes through SEs in mESCs. Taken together, PCGF6 activates the pluripotency genes in mESCs by binding to their upstream SEs.

### PCGF6 is recruited into a subset of SE regions by OCT4

**Figure 4:**
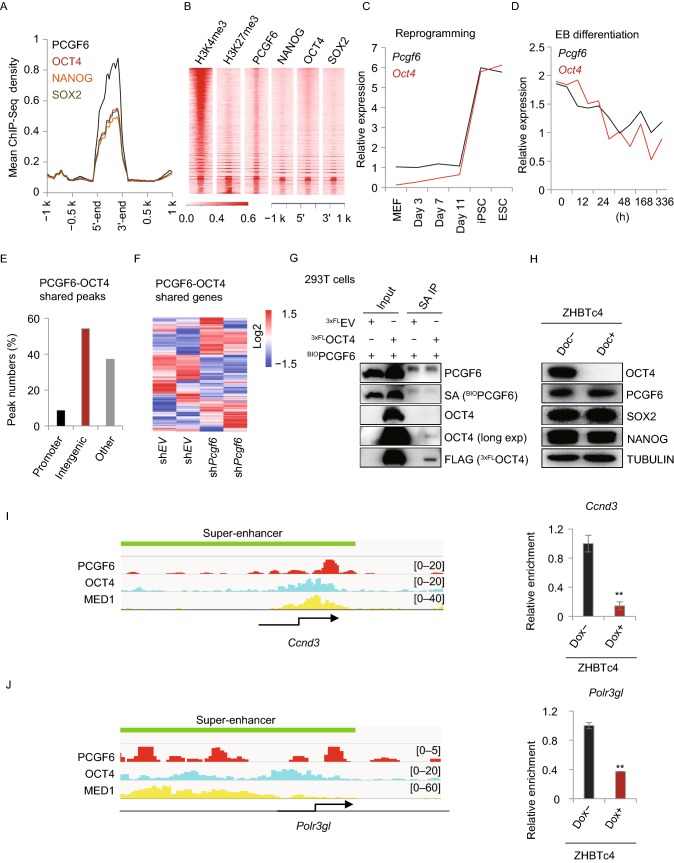
**Role of OCT4 and PCGF6 in Super-enhancer Regions**. (A) Average ChIP-Seq density of OCT4, SOX2 and NANOG near the PCGF6 peak center. (B) Heatmaps of PCGF6 binding loci that are sorted by H3K4me3 and H3K27me3, and the distribution of OCT4, SOX2 and NANOG at PCGF6 binding sites. (C and D) The relative expression pattern of PCGF6 and OCT4 during somatic cell reprogramming (C) and EB differentiation (D). (E) Distribution of PCGF6/OCT4 co-binding sites at promoter (−3 kb to +3 kb), intergenic, and other regions. (F) Relative expression of PCGF6/OCT4 co-binding genes in *Pcgf6*-depleted group compared with empty vector group. (G) Validation of physical associations of PCGF6 and OCT4 in 293T cells by co-immunoprecipitation. (H) Protein levels after 23 h of dox treatment in ZHBTc4 mESCs. (I–J) The enrichment of PCGF6, MED1 and OCT4 at the SE region of *Ccnd3* and *Polr3gl*. Dox treatment decreased the enrichment of PCGF6 at the SEs of *Ccnd3* and *Polr3gl*. Data are presented as mean ± SD from three independent replicates. **P < 0.01 compared with control cells

Since SEs are usually co-occupied by multiple core pluripotency factors, including OSN (Kagey et al., 2010; Whyte et al., 2013), we hypothesized that PCGF6 co-localizes with these factors to activate the transcriptome through SEs in mESCs. Indeed, we analyzed the enrichment of OSN in PCGF6 target regions, and found that all the three core pluripotency factors were enriched in the PCGF6 binding regions (Fig. 4A). Interestingly, OCT4 was preferably enriched in the binding regions of PCGF6 (Fig. 4B). Then we focused on the correlation between OCT4 and PCGF6. Consistent with this observation, both PCGF6 and OCT4 displayed similar expression patterns during somatic cell reprogramming and EB differentiation (Fig. 4C and 4D), indicating a synergistic action in regulating cell fate decision. Therefore, we hypothesized PCGF6 coordinates with OCT4 to regulate gene expression by SEs. We analyzed the distribution of the co-binding regions between PCGF6 and OCT4 (Fig. 4E). The results showed that about 54.2% of these co-binding sites were in the intergenic regions, 37.2% of the sites were distributed in gene body and other regions, and only 8.6% of these sites located in promoter regions (Fig. 4E). Intriguingly, OCT4 preferably bound to the active regions of PCGF6 (70.5%) rather than the repressive regions (51%) (Fig. S3A), and MED1 showed the significant binding (78.5%) at the PCGF6-OCT4 co-regions (Fig. S3B). Since the SEs are mostly enriched in the intergenic regions and enriched for OCT4, it suggested an SE dependent regulation pattern between PCGF6 and OCT4 (Fig. 4E).

Based on the findings above, we next analyzed the transcriptome dynamic of those genes co-occupied by PCGF6 and OCT4. Importantly, a large number of these genes were downregulated after *Pcgf6* knockdown (Fig. 4F; Table S5), indicating that PCGF6 activates gene expression with the participation of OCT4. Besides, co-immunoprecipitation (co-IP) of PCGF6 and OCT4 further confirmed a physical interaction between them (Fig. 4G). To further illuminate their relationship, we treated ZHBTc4 cells (murine ESC line) with doxycycline (dox) for 23 h, and found that dox-induced conditional knockout of OCT4 in the ZHBTc4 cells did not significantly affect the levels of the SOX2, NANOG and PCGF6 proteins (Fig. 4H), indicating that the pluripotent phenotype was retained. Chromatin immunoprecipitation coupled with quantitative real-time PCR (ChIP-qPCR) analysis showed that the enrichment of PCGF6 at the *Ccnd3* and *Polr3gl* loci decreased significantly after dox treatment (Fig. 4I and 4J) despite the normal protein levels of PCGF6 (Fig. 4H). We have also detected more targets besides *Ccnd3* and *Polr3gl*, and the results were consistent with the above (Fig. S3C). Therefore, these results indicate that PCGF6 functions as an activator of regulating gene expression through SEs in mESCs, and OCT4 recruits PCGF6 to a subset of SEs to activate their downstream gene expression. Taken together, our results provide a novel regulatory mechanism that PCGF6 and OCT4 target SE regions to activate pluripotency genes.

### PCGF6 coordinates with OCT4 to mediate SE/promoter interactions via 3D chromatin

**Figure 5:**
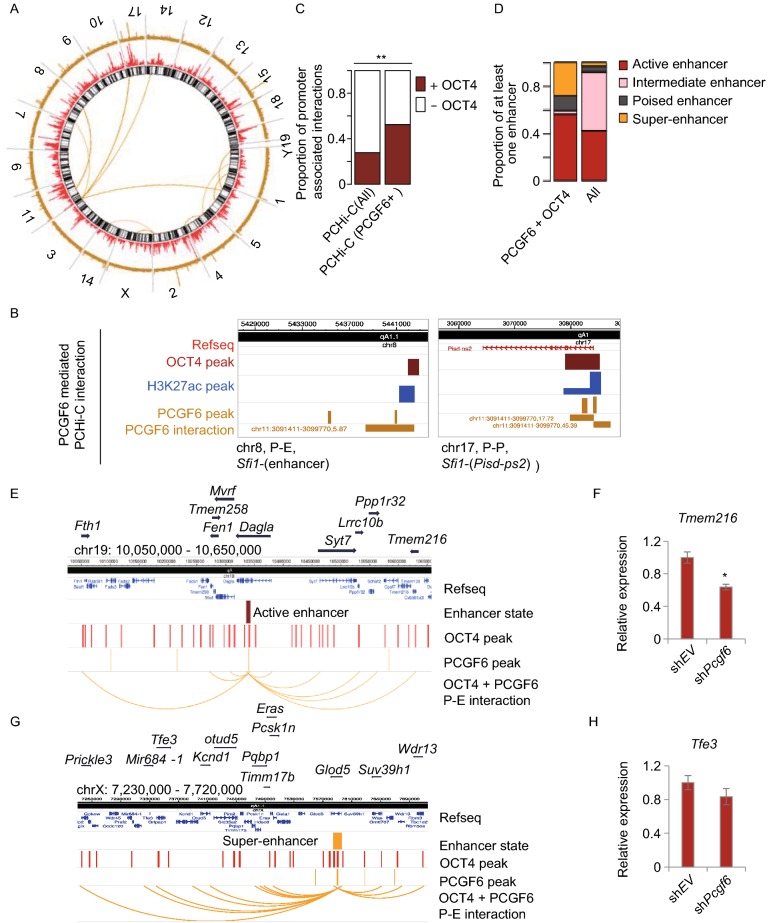
**Role of OCT4 and PCGF6 in 3D chromatin**. (A) Circos diagram of genomic promoter associated interactions regulated by PCGF6. The black color refers to gene position. The red color is Refseq genes and refers to gene density. The yellow color refers to intra-promoter-promoter interactions. The orange color refers to inter-promoter-promoter interactions. (B) Promoter-associated interactions mediated by PCGF6 at *Sfi1* promotor. The promoter interactions of *Sfi1* with enhancer and promoter are regulated by PCGF6 and OCT4. (C) The percentage of OCT4 in promoter-associated interactions regulated by PCGF6 or in all promoter associated interactions. **P < 0.01 compared with control group. (D) The percentage of promoter-associated interactions regulated by active enhancer, intermediate enhancer, poised enhancer, and super-enhancer co-occupied by PCGF6 and OCT4. (E–H) Promoter-associated interactions of *Tmem216* (E) and *Tfe3* (G) derived from promoter capture Hi-C reads, and relative expression of *Tmem216* (F) and *Tfe3* (H) in *Pcgf6*-depleted group compared with empty vector group. The regions and genes above are closest to SE region regulated by PCGF6. Data are presented as mean ± SEM (*n* = 3). *P < 0.05 compared with control cells

Enhancer-promoter interactions occur predominantly via forming 3D chromatin structure, which are important for driving expression of the adjacent genes in pluripotent cells (Ji et al., 2016). Recent study showed that the other PcG subunits like EED and RING1B play indispensable roles in the formation of extremely long-range promoter-promoter interactions (ELRIs) (Joshi et al., 2015). To determine whether PCGF6 plays a similar role in regulating the 3D chromatin structure, promoter capture Hi-C data was used to analyze the promoter-associated interaction in the genome-wide. By mapping the PCGF6 peaks from ChIP-Seq data with the identified promoter associated interaction regions, we found that PCGF6 participated in promoter-associated interactions in 3D chromatin (Fig. 5A). These promoter-associated interactions were classified into the promoter-promoter interactions and promoter-enhancer interactions, with the former being more frequent (Fig. S4A). Some genes like *Sfi1* were regulated by both enhancer-promoter and promoter-promoter interactions (Fig. 5B). Since OCT4 can influence the enrichment of PCGF6 at the co-binding sites, we next analyzed whether OCT4 also influenced these promoter-associated interactions mediated by PCGF6. As shown in Fig. 5C, OCT4 preferably participated in promoter-associated interactions mediated by PCGF6 via the co-occupied enhancer regions compared with all promoter-associated interactions (Fig. 5C). These enhancers including active enhancers, SEs, intermediate enhancers and poised enhancers, respectively characterized by H3K4me3, H3K27me3 and H3K4me1 (Fig. 5D) (Schoenfelder et al., 2015). Interestingly, PCGF6 and OCT4 preferably bound together to active enhancers and SEs (Fig. 5D). For examples, PCGF6 and OCT4 regulated the *Tmem216* promoter via binding to the distal active enhancer (Fig. 5E), and *Tmem216* was downregulated when *Pcgf6* depletion (Fig. 5F). Furthermore, PCGF6 and OCT4 were also enriched in the SE region, which regulates the distal *Tfe3* promoter (Fig. 5G), which was also downregulated in the event of *Pcgf6* depletion (Fig. 5H). Taken together, PCGF6 coordinates with OCT4 to participate in 3D chromatin interactions, and then regulate gene expression through enhancers/promoter interactions.

### PCGF6 coordinates with OCT4 to regulate a subset of proliferation genes through the SEs

**Figure 6:**
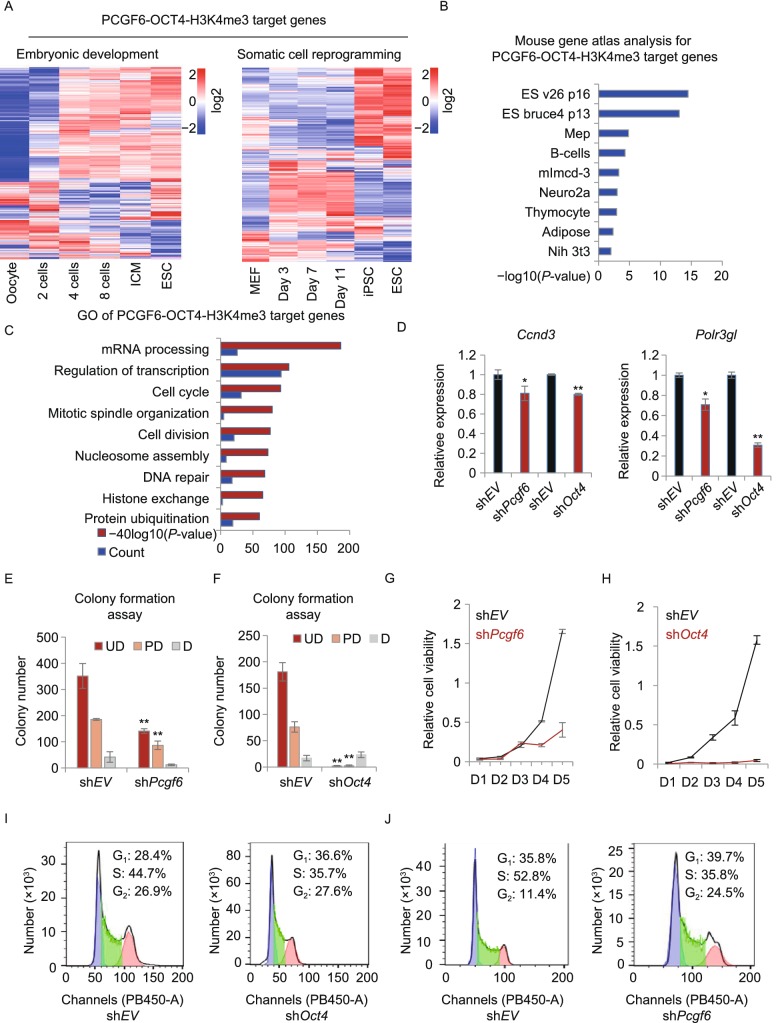
**PCGF6 coordinates with OCT4 in regulating self-renewal of mESCs**. (A) Heatmaps shows the dynamic expression of PCGF6/OCT4/H3K4me3 co-binding genes during embryonic development and somatic cell reprogramming. (B) Mouse gene atlas analysis of PCGF6/OCT4/H3K4me3 co-binding genes enriched in in different tissues and cell lines. (C) Gene ontology analysis of PCGF6/OCT4/H3K4me3 co-binding genes. (D) Relative expression of cell cycle genes *Ccnd3* and *Polr3gl* in *Pcgf6*-depleted group or *Oct4*-depleted group compared with empty vector group. Data are presented in three independent replicates. **P < 0.01 compared with control cells. *P < 0.05 compared with control cells. (E and F) Knockdown of *Pcgf6* (E) and *Oct4* (F) significantly repressed colony formation of mESCs. Data are presented as mean ± SD from three independent replicate experiments. **P < 0.01 compared with control cells. (G and H) Cell proliferation was evaluated by cell counting kit 8 cell viability assay. Data are presented as means ± SD from three independent replicate experiments. (I–J) Distribution of cell population in G_1_, S and G_2_ phase in *Pcgf6*-depleted group, *Oct4*-depleted group and empty vector group. Cells were stained by DAPI. Blue, green and red represent the cell stage of G_1_, S and G_2_, respectively. The experiments were performed in triplicate

To further demonstrate the coordinating action of PCGF6 and OCT4 in mESCs, we analyzed the expression of the genes activated by both during embryonic development and somatic cell reprogramming, and observed their high expression in both situations (Fig. 6A). Furthermore, a subset of these genes were upregulated from day 3 to day 11 of somatic cell reprogramming, indicating an important role of PCGF6 in establishing pluripotency (Fig. 6A). To identify the roles of these genes, analysis of the mouse gene atlas showed that these genes co-activated by PCGF6 and OCT4 are highly expressed in ESC lines relative to other tissues (Fig. 6B). In addition, the Gene Ontology analysis demonstrated a significant enrichment in the processes of high transcription and cell cycle process (Fig. 6C). Cell cycle process regulated by cell cycle associated genes was characterized by a short G_1_ phase, which served to limit the potential for differentiation because cells preferentially initiate differentiation from the G_1_ phase (White and Dalton, 2005; Singh and Dalton, 2009). As examples, cell cycle genes *Ccnd3* and *Polr3gl* were downregulated after *Pcgf6* or *Oct4* knockdown (Fig. 6D), and these genes were regulated through the SEs targeted by PCGF6. We experimentally validated the proliferative effects of PCGF6 and OCT4 in the *Pcgf6* or *Oct4* knockout cell lines respectively, and found that absence of either significantly reduced the viability and colony forming ability of the cells (Figs. 6E–6H and S5A). Furthermore, *Oct4* and *Pcgf6* knockdown in mESCs dramatically decreased the S-phase cell population but increased that of G_1_- or/and G_2_-phase (Figs. 6I, 6J and S5B), suggesting a slowing down of the proliferation rates after *Oct4* and *Pcgf6* knockdown. To further validate our results, we overexpressed *Ccnd3* and *Polr3gl* in the *Pcgf6*-depleted cells (Fig. S5C), and found that the proliferation rate was restored (Fig. S5D), as were the expression of pluripotency genes like *Rest*, *Tfcp2l1*, *Zfp281*, *Oct4*, *Lin28a* and *Esrrb* (Fig. S5E). In conclusion, our results showed that PCGF6 and OCT4 co-bind to the SEs of a subset of proliferation-related genes in mESCs to regulate cell cycle progression.

## Discussion

**Figure 7:**
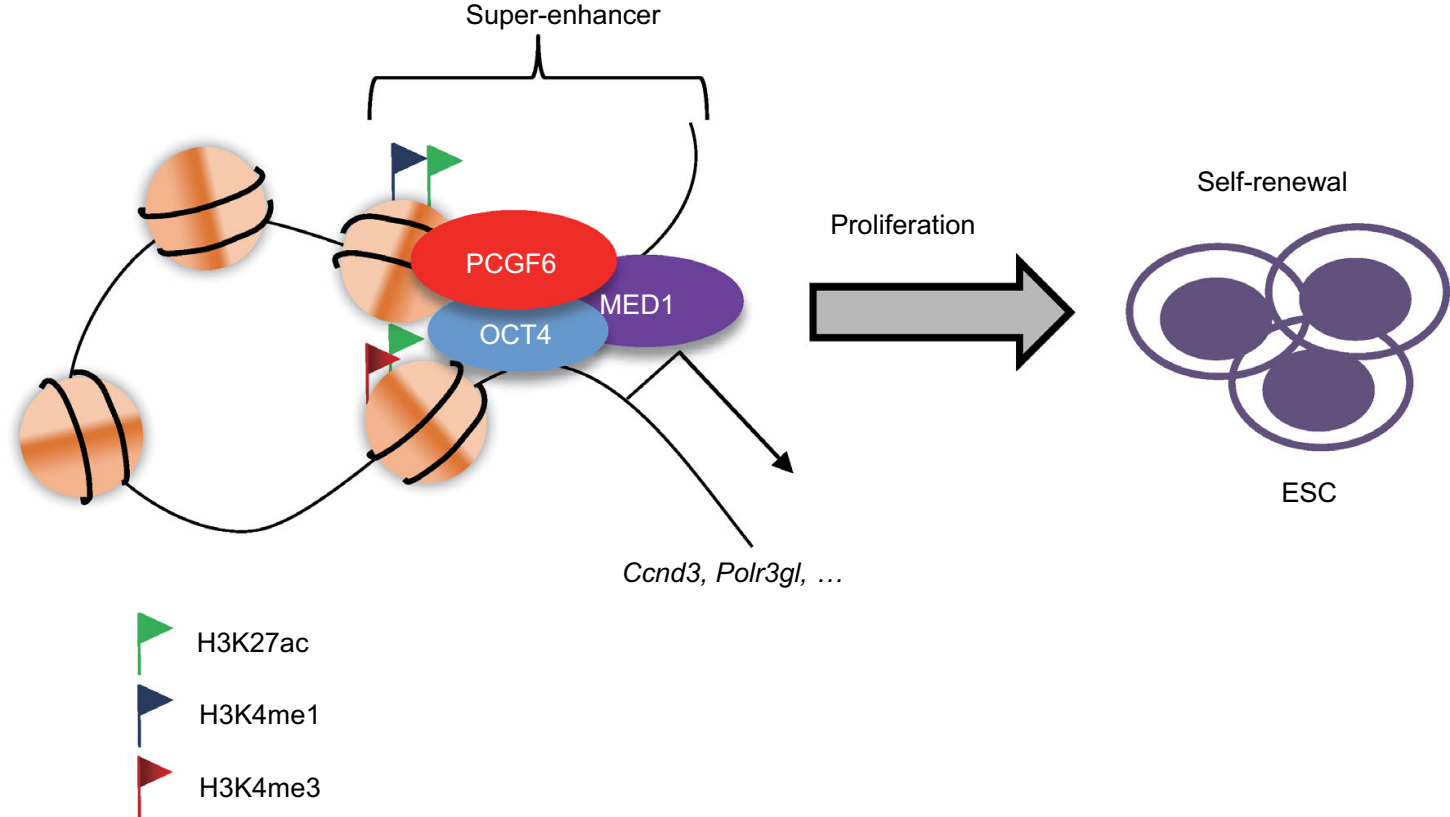
**The functional model of PCGF6 and OCT4**. In this model, PCGF6 function as a transcription activator for pluripotency in mESCs. In detail, PCGF6 activates cell cycle gene including *Ccnd3* and *Polr3gl* via the super-enhancer dependent chromatin Interactions to promote mESCs proliferation, meanwhile, OCT4 is required for the binding of PCGF6 at these transcription activation regions

Our study aims to study the mechanism of PCGF6 function as a transcription activator in mESCs. The significantly high expression of *Pcgf6* in ESCs strongly suggests an important role in maintenance of pluripotency. In this study, we identified PCGF6 as an important factor for ESC self-renewal and late stage pre-iPSC reprogramming, and showed that PCGF6 promotes the expression of ESC proliferation genes and maintains self-renewal through SE-dependent chromatin interactions by interacting with OCT4 (Fig. 7).

Here we proposed a vital role for *Pcgf6* in pre-iPS cell reprogramming. Knockdown of *Pcgf6* significantly reduced the reprogramming efficiency, and the expression of *Pcgf6* is the highest in the later stage of somatic cell reprogramming, which indicate that PCGF6 regulates pluripotency establishment specifically in the later stage of reprogramming. A recent study reported that PCGF6 can replace SOX2 during somatic cell reprogramming and knockdown of *Pcgf6* significantly decreases iPSC colony numbers (Zdzieblo et al., 2014). Ectopic expression of *Pcgf6* dramatically increased the number of *Oct4*-GFP colonies (Yang et al., 2016), which was consistent with our experimental results that the *Oct4*-GFP colony numbers decreased by more than 50% after knockdown of *Pcgf6* in pre-iPS cell reprogramming. These results verified a functional role of PCGF6 in the regulation of the pluripotency network, especially in the regulation of pluripotency establishment in the later stage of reprogramming.

PcG proteins are known to repress gene expression via chromatin remodeling, and PRC1 and PRC2 functionally modify histones by recruiting specific PCGF components (Gao et al., 2012; Endoh et al., 2017). Based on the PCGF family member,, PRC1 complexes can be classified into six groups, PRC1.1–1.6, distinguished by the presence of a different member of the PCGF family (Schwartz and Pirrotta, 2013). PCGF6, a PRC1.6 component, is enriched in the SE regions and acts as a transcriptional activator for the pluripotency genes. Although PCGF6 is enriched in only a subset of SEs, *Pcgf6* knockdown downregulated a majority of SE-associated genes, including *Oct4*. Moreover, since SEs are highly enriched for many pluripotency factors, it is possible that the downstream genes activated by PCGF6 are probably co-binding with other pluripotency factors other than OCT4. From the aspect of epigenetics, ChIP-Seq data shows that TrxG have a co-localization with PCGF6 and H3K4me3. Intriguingly, previous evidence demonstrates that the genomic distributions of OCT4 and WDR5 localization were strikingly similar (Ang et al., 2011). The work above suggests that the activation sites targeted by PCGF6 is probably also enriched for TrxG. Importantly, PcG regulate the extremely long-range intra- and inter-chromosomal interactions in bivalent promoters involving the *Hox* clusters in 3D chromatin structure (Joshi et al., 2015). Similarly, TrxG also mediated 3D chromatin interaction (Mas et al., 2018) . Therefore, there may be a possibility that PCGF6, a PcG component, regulates 3D chromatin interaction with either PcG or TrxG. Besides, PCGF6 is involved in both promoter-promoter and enhancer-promoter interaction according to promoter capture Hi-C data. Nevertheless, whether PCGF6 mediates 3D chromatin interaction or functions as a structural factor to maintain the 3D structure is unclear. It is worthwhile further analyzing the specific regulation mechanism of PCGF6 in 3D chromatin.

Taken together, our study for the mechanism of PCGF6 activating transcriptome further consolidates the vital role of PCGF6 in pluripotency network. The mechanism how PCGF6 regulates stem cell pluripotency is still not clear, future studies will be required. Furthermore, the study of PCGF6 will shed new light on novel PcG function and provide additional methods to regulate the efficiency of programming. These provide a research basis for therapeutic application of pluripotent stem cells.

## Materials and methods

### Cell culture

R1 mESCs, ZHBTc4 (mESC lines) (Niwa et al., 2000) and pre-iPS cells were cultured on gelatin-coated tissue culture plates in knockout DMEM (Thermo Fisher, 10829018) supplemented with 15% fetal calf serum (Lonsera, S711-004S/NN02953), 1× nonessential amino acids (Gibco, 11140050), 2 mmol/L L-glutamine (Gibco, 35050061), 1% (*v*/*v*) nucleoside mix (Sigma-Aldrich, A-4036, T-1895, C-4654, G-6264, U-3003), 0.1 mmol/L β-mercaptoethanol (Sigma-Aldrich, M6250), 1,000 U/mL recombinant leukemia inhibitory factor (LIF) (Millipore, ESG1107).

### Alkaline phosphatase staining

R1-mESCs and iPSCs derived from pre-iPS cells were fixed and stained for alkaline phosphatase activity using an alkaline phosphatase staining kit (Sigma-Aldrich, 86R-1KT). Plates are fixed by adding citrate-acetone-formaldehyde fixative solution for 30 s, and are rinsed gently in deionized water for 45 s. Then alkaline-dye mixture is added to plates at 18–26 °C for 15 min in dark room. After 15 min of incubation, dye mixture will be removed from plates and rinse the plates for 2 min in deionized water, air dry the plates, and then evaluate and record the colonies in the plates microscopically.

### Colony-formation assay

On the fifth day after the infection of empty vector (sh*EV*) and *Pcgf6* knockdown (sh*Pcgf6*) or *Oct4* knockdown (sh*Oct4*) lentivirus, 1,000 mESCs were seeded into individual wells of a 6-well plate. The cell colonies were stained for alkaline phosphate after 5 days of culture. Colonies of differentiated cells (D), undifferentiated cells (UD) and partially differentiated cells (PD) in each well of 6-well plate were scored respectively. Experiments were performed in triplicates.

### Cell cycle analysis

R1 mESCs with *Pcgf6* or *Oct4* knockdown were obtained 5 days after lentivirus infection. Each group of cells was collected 4 or 5 days after infection and fixed in 70% ethanol overnight. Cells were washed three times with PBS, and then stained by the DAPI solution (3 μmol/L DAPI/0.1% Triton X-100 in PBS) for 15 min at room temperature. Cells were analyzed by flow cytometry (CytoFLEX, Beckman Coulter, US). DAPI signal was collected with the PB450 channel. Experiments were performed in triplicate.

### Cell viability assay

R1 mESCs with *Pcgf6* or *Oct4* knockdown were obtained 5 or 4 days after lentivirus infection. 500 cells for each group suspend in 100 µL mESC medium were added to individual wells of a 96-well plate coated with gelatin. Then cells will be incubated overnight. Each well was then supplemented with 10 µL cell counting kit 8 (CCK8) (Dojindo, JP) followed by incubation at 37 °C for 3 h, and then analyzed absorbance of 450 nm. The viable cells in each group were detected for five consecutive days. Experiments were performed in triplicate and results shown as the means ± SD.

### Chromatin immunoprecipitation (ChIP) coupled with quantitative real-time PCR (ChIPqPCR)

ChIP was performed as previously described (Lee et al., 2006). The primers were designed according to PCGF6 peaks or PCGF6/OCT4 shared peaks. Real-time PCR was performed with a LightCycler 480 (Roche) instrument using the SYBR qPCR Master Mix reagents (Vazyme, Q711-00). Differences between samples and controls were calculated based on the 2-∆CT method and normalized to Input. Measurements were performed in triplicate. Primers used are listed in Table S1.

### Vector construction

The pLKO vector is modified from pLKO.1 (Addgene, 8453) (Stewart et al., 2003). We designed three shRNA sequences for each gene, and selected the one with highest knockdown efficiency by qPCR and Western blot to perform the next functional studies. The shRNA sequences to efficiently target *Pcgf6* and *Oct4* is CTGATAGATGCAACCACCATT and CCTACAGCAGATCACTCACAT respectively.

### RNA extraction and real time quantitative PCR (qPCR)

Total RNA was extracted using the Eastep® Super Total RNA Extraction Kit (Promega, LS1040) and cDNA was generated using PrimeScript™ RT Master Mix (Takara, RR036A). Relative expression were determined using the SYBR qPCR Master Mix (Vazyme, Q711-00) on LightCycler 480 system (Roche). Differences between experimental group and control group were calculated based on the 2 ^ (-∆∆Ct) method and normalized to *Gapdh*. Measurements were performed in triplicate in every independent experiment. Primers used are listed in Table S1.

### Reprogramming assays in pre-iPS cells

Pre-iPS cells were constructed in our previous work (Costa et al., 2013). In detail, these cells derived from adult neural stem (NS) cells infected with pMX-based retroviral reprogramming factors OCT4, KLF4 and c-MYC (OKM) and cultures were switched to ES cell medium (serum/LIF) at day 3 post-transduction. The clonal lines of reprogramming intermediates transgenic for PB-flox-Nanog-Pgk-Hygro were infected by the Pcgf6 knockdown lentiviruses. After 4 days of selection by 35 µg/mL Blasticidin, 1 × 10^5^ cells were seeded into a 12-well plate in serum/LIF medium with 20 µg/mL Blasticidin and 150 µg/mL Hygromycin. After 2 days later, medium was switched to N2B27/2i/LIF with 20 µg/mL Blasticidin and 150 µg/mL Hygromycin (Silva et al., 2008). GFP-positive colonies were scored microscopically at day 10 after medium switch, which were performed in triplicate.

### Co-immunoprecipitation (co-IP) and Western blot analysis

Cell extracts were prepared from 293T cells. Exogenous PCGF6 was immunoprecipitated with 7.5 µL streptavidin agaroses (Novex by Lifetechnologies, 15942-050) for each sample, and co-immunoprecipitated PCGF6 was identified by Western blot with a Streptavidin-HRP (GE Healthcare Life sciences Amersham, RPN1231), OCT4 antibody (Santa Cruz, sc5279), FLAG antibody (Sigma, F1804).

### Total Protein extract preparation and Western blot analysis

Total protein was extracted using the CyterBuster Extract Buffer with 1× protein inhibitor cocktail. Then samples identified by Western blot with a RING6A antibody (Abcam, ab200038), OCT4 antibody (Santa Cruz, sc5279), SOX2 antibody (Stemgent, 09-0024), NANOG antibody (Bethyl, A300-397A). TUBULIN antibody (Abcam, ab6046) and β-actin antibody (Sigma, A5441) were used as the loading control in this study.

### ChIP sequencing analysis

Gene Interval Notator [GIN, (37)] was used to annotate peaks over Refseq mouse genes. Promoters were defined as 6 kb regions (±3 kb) surrounding the transcriptional start site (TSS). A peak was generated by the TSS of a Refseq gene that falling into the surrounding 6 kb (±3 kb). Datasets are available for download from NCBI’s Gene Expression Omnibus (GEO, http://www.ncbi.nlm.nih.gov/geo). ChIP-Seq data and relative negative controls were download according to the GEO accession numbers that listed in Table S2.

### RNA sequencing analysis

Two replicates of RNA-Seq for empty vector control (sh*EV*) and *Pcgf6* depletion (sh*Pcgf6*) of mESCs were performed respectively. We prepared about 6ug total RNA for each sample to sequence with the Illumina high-throughput sequencing platform, with a poly-A selection method. A total of 30–40 million single end reads were generated for each sample. Adapter of reads were trimmed to the genome using Trim Galore (v 0.5.0), and then the remaining reads are mapped to the genome using Star2. The number of reads matched on each gene will be calculated. A change in gene expression after knockdown of Pcgf6 higher than 1.3 folds and *P* < 0.05 was significantly. *P*-value was calculated to investigate the reliability among the genes expression changes caused by knockdown of *Pcgf6*. Genes that are differentially expressed listed in Table S3.

### Accession number

Affymetrix gene expression profile (GEP) data of embryonic development (GSE22182) and somatic cell reprogramming (GSE19023) were analyzed by the “affy” package (v3.1.2) from Bioconductor and normalized by the RNA algorithm, and then the probes were matched to corresponding gene symbol by certain platforms.

### Functional annotations

Gene Ontology Biological Processes analysis and Mouse Gene Atlas analysis were generated using DAVID Bioinformatics Resources 6.8 (https://david.ncifcrf.gov/).

### Promoter capture Hi-C (PCHi-C) analysis

Raw PCHi-C, Hi-C and random ligation control data in mESCs are downloaded from NCBI’s Gene Expression Omnibus under accession number GSE81503 (GEO, http://www .ncbi.nlm.nih.gov/geo). The reads were mapped to the genome wide DNA sequences interacting with promoters against the mouse (mm9). The HiCUP pipeline was used to process the reads. The resulting BAM files were processed into CHiCAGO input files, retaining only those read pairs that mapped two end to a captured bait. A 95% confidence interval for the overlap was obtained from 100 random draws.

## Electronic supplementary material

Below is the link to the electronic supplementary material.

Supplementary material 1 (PDF 195 kb)

Supplementary material 2 (XLSX 15 kb)

Supplementary material 3 (XLSX 11 kb)

Supplementary material 4 (XLSX 169 kb)

Supplementary material 5 (XLSX 41 kb)

Supplementary material 6 (XLSX 137 kb)
