# Supplementary material for: PCGF6 regulates stem cell pluripotency as a transcription activator via super-enhancer dependent chromatin interactions
Source: Protein Cell. 2019 Apr 30;10(10):709–25. doi: 10.1007/s13238-019-0629-9 (PMC6776568; doi:10.1007/s13238-019-0629-9)

Supplemental Information

PCGF6 Regulates Stem Cell Pluripotency as a Transcription Activator via Super-Enhancer Dependent Chromatin Interactions

Xiaona Huang, Chao Wei, Fenjie Li, Lumeng Jia, Pengguihang Zeng, Jiahe Li, Jin Tan, Tuanfeng Sun, Shaoshuai Jiang, Jia Wang, Xiuxiao Tang, Qingquan Zhao, Bin Liu, Limin Rong, Cheng Li, and Junjun Ding

Supplemental Figures

Figure S1. PCGF6 maintains mESC identity. Related to Figure 1. (A-E) Heatmaps of the marker gene expression for pluripotency (A), Trophectoderm (B), Mesoderm (C), Ectoderm (D), and Endoderm (E) by using RNA-Seq data from sh*EV* and sh*Pcgf6* knockdown ESCs. The scale represents fold changes.

Figure S2. PCGF6 regulates SE associated genes. Related to Figure 3. (A) Overlapping of the targeted regions of PCGF6/OCT4, PCGF6/SOX2 and PCGF6/NANOG. (B) Overlapping of the SE associated genes with downregulated or upregulated genes after *Pcgf6* knockdown, respectively. (C) Relative expression of SE associated genes were significantly downregulated or upregulated after *Pcgf6* knockdown. The scale represents $\log_2$ (fold change).

Figure S3. PCGF6 coordinates OCT4 in regulating SEs. Related to Figure 4. (A) Percentage of OCT4 binding to PCGF6/H3K4me3 or PCGF6/H3K27me3 target genes, respectively. (B) Percentage of MED1 binding to PCGF6/OCT4 shared regions. (C) Depletion of OCT4 by dox treatment decreased the enrichment of PCGF6 at the SEs of *Oct4*, *Sox2*, *Nanog*, *Klf4*, *Pim1*, and *Agtrap*. Data are presented as mean ± SD from three independent replicates. **$p < 0.01$ compared with control cells. *$p < 0.05$ compared with control cells.

Figure S4. PCGF6 and OCT4 participate in 3D chromatin. Related to Figure 5. Percentage of promoter-promoter or promoter-enhancer interactions regulated by PCGF6.

Figure S5. PCGF6 and OCT4 regulate proliferation genes. Related to

Figure 6. (A) Colony formation assay for ESCs after *Pcgf6* or *Oct4* knockdown. Individual colonies were stained for AP activity. (B) Cell cycle analysis for ESCs after *Pcgf6* or *Oct4* knockdown. **p < 0.01 compared with control cells. *p < 0.05 compared with control cells. (C) Overexpression efficiency of *Ccnd3* and *Polr3gl* in mESCs was validated by Real-Time Quantitative PCR (qPCR). Data are presented as mean ± SD from three independent replicates. **p < 0.01 compared with sh*EV*+*EV* cells. *p < 0.05 compared with sh*EV*+*EV* cells. (D) Cell proliferation was evaluated by cell counting kit 8 cell viability assay. Data are presented as means ± SD from three independent replicate experiments. The experiments were performed in triplicate. (E) Expression changes of pluripotency genes in response to knockdown of *Pcgf6* were rescued by introduction of *Ccnd3* or *Polr3gl*. Data are depicted as mean ± SD from three independent replicates. **p < 0.01 compared with sh*EV*+*EV* cells. *p < 0.05 compared with sh*EV*+*EV* cells. $^{\#\#}$p < 0.01 compared with sh*Pcgf6*+*EV* cells. $^{\#}$p < 0.05 compared with sh*Pcgf6*+*EV* cells.

Supplemental Tables

Table S1. Primers used in this study for RT-qPCR and ChIP-qPCR.

Table S2. Published GEO datasets used in this study.

Table S3. RNA-Seq data on genes that are differentially expressed after 5 days of Pcgf6 KD.

Table S4. List of PCGF6 target genes used in this study.

Table S5. List of OCT4 target genes used in this study

Supplement figure 1

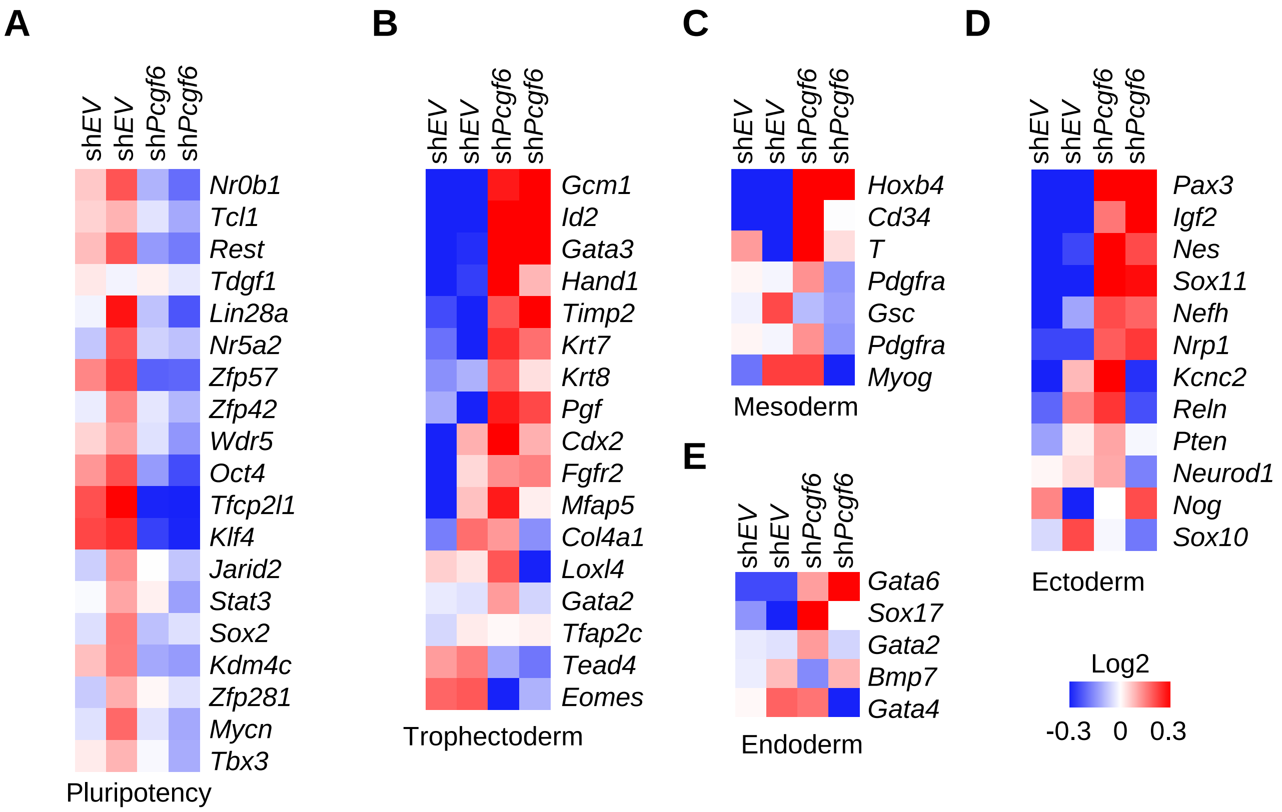

Supplement figure 2

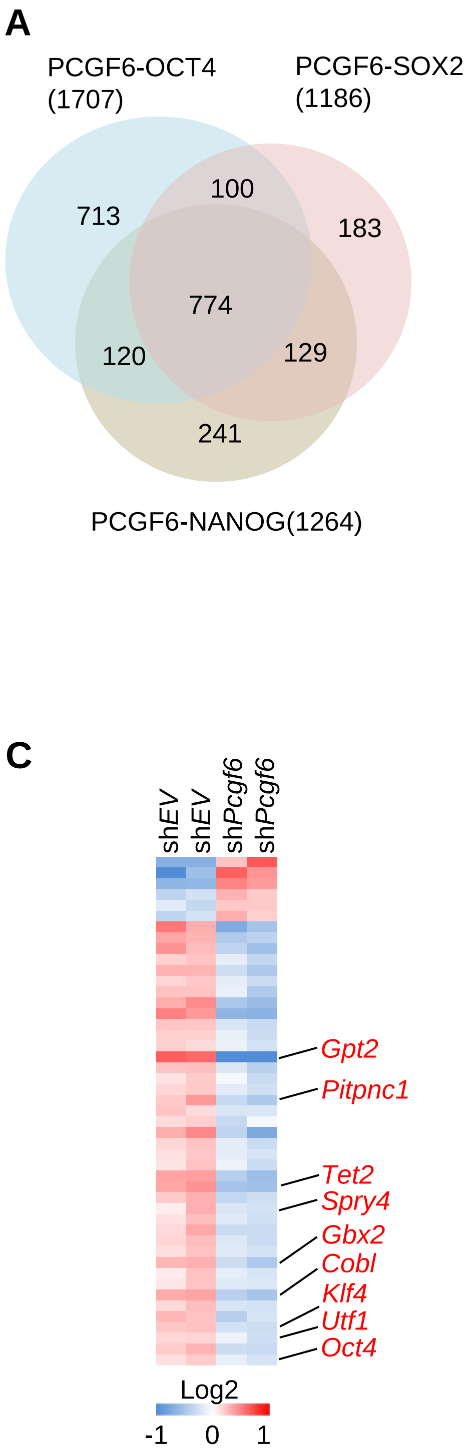

Supplement figure 3

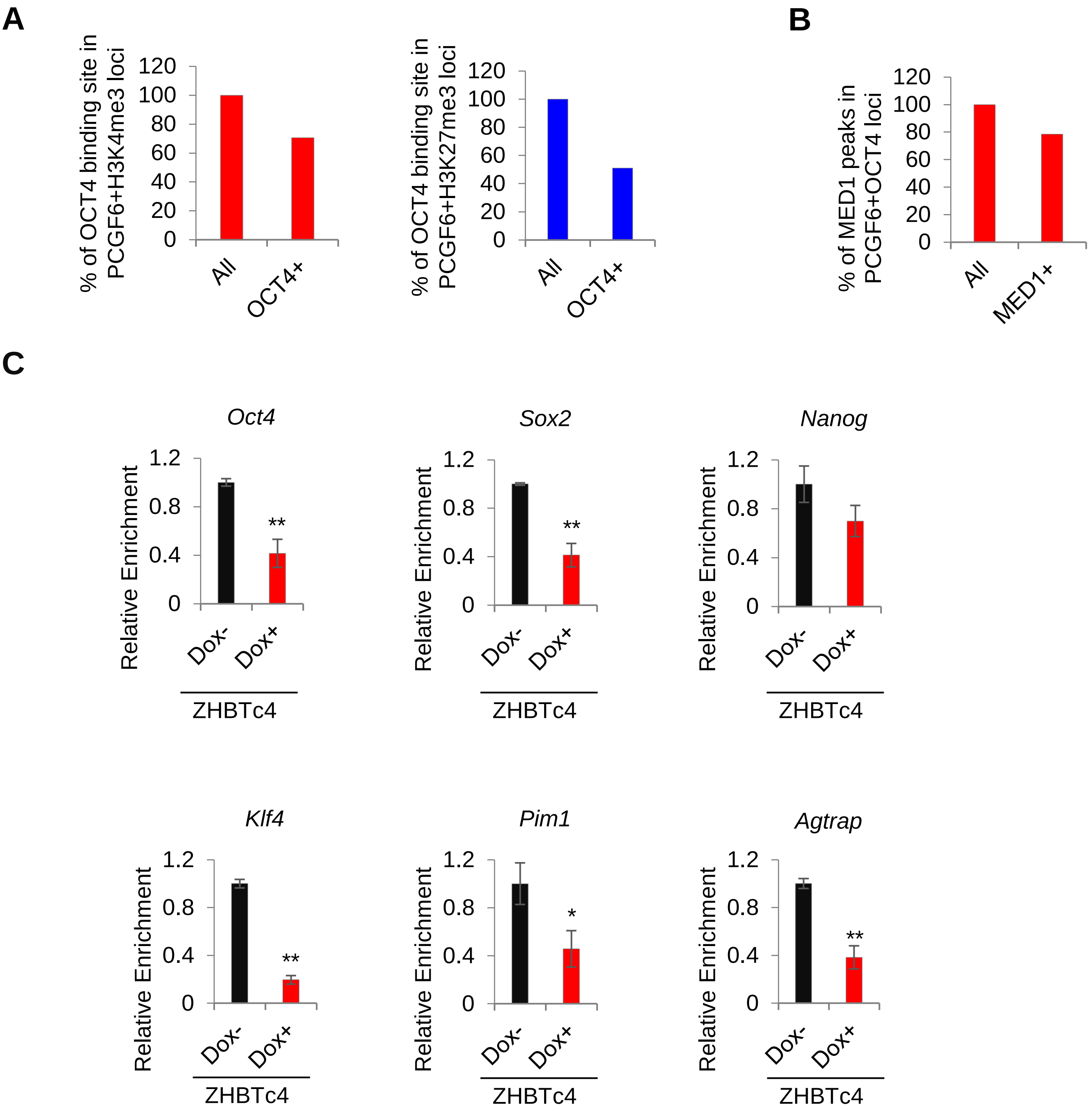

Supplement figure 4

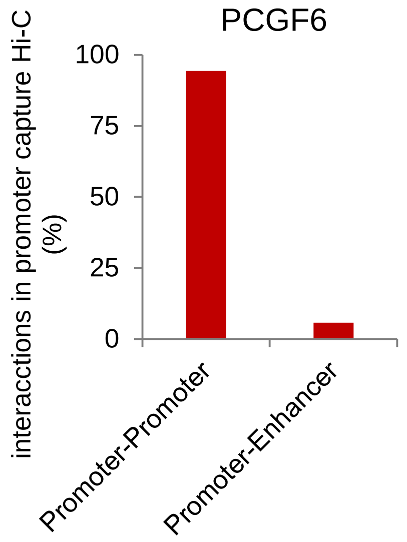

Supplement figure 5

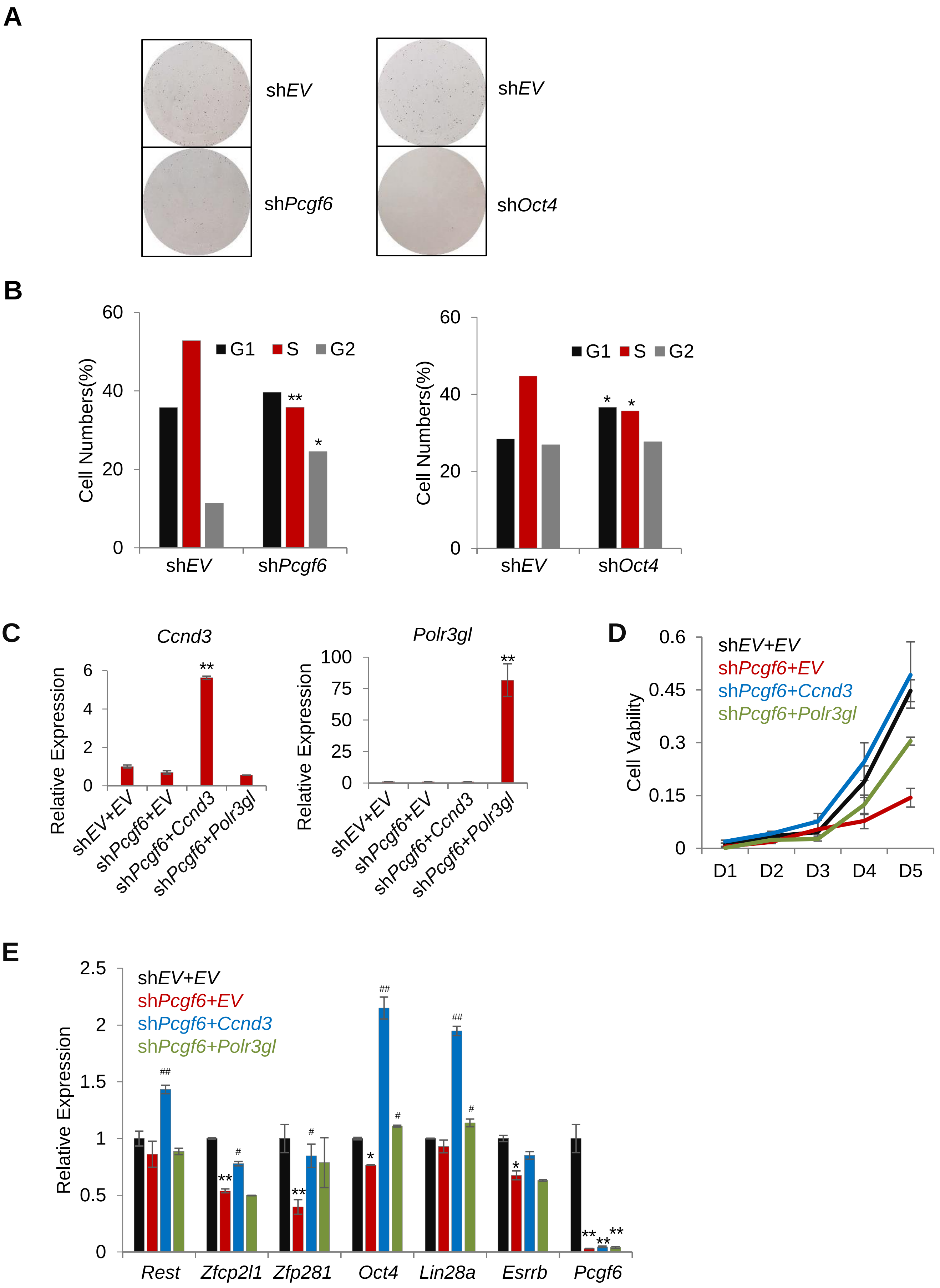

Supplement: Supplementary file 1 — Supplementary material 1 (PDF 195 kb) [file 13238_2019_629_MOESM1_ESM.pdf]
